# Porous Boron Nitride Materials: Influence of Structure, Chemistry and Stability on the Adsorption of Organics

**DOI:** 10.3389/fchem.2019.00160

**Published:** 2019-03-26

**Authors:** Sofia Marchesini, Xiyu Wang, Camille Petit

**Affiliations:** Department of Chemical Engineering, Barrer Centre, Imperial College London, London, United Kingdom

**Keywords:** boron nitride, vapor sorption, adsorption, separations, water stability

## Abstract

Porous boron nitride (BN) is structurally analogous to activated carbon. This material is gaining increasing attention for its potential in a range of adsorption and chemical separation applications, with a number of recent proof-of-concept studies on the removal of organics from water. Today though, the properties of porous BN—i.e., surface area, pore network, chemistry—that dictate adsorption of specific organics remain vastly unknown. Yet, they will need to be optimized to realize the full potential of the material in the envisioned applications. Here, a selection of porous BN materials with varied pore structures and chemistries were studied for the adsorption of different organic molecules, either directly, through vapor sorption analyses or as part of a water/organic mixture in the liquid phase. These separations are relevant to the industrial and environmental sectors and are envisioned to take advantage of the hydrophobic character of the BN sheets. The materials were tested and regenerated and their physical and chemical features were characterized before and after testing. This study allowed identifying the adsorption mechanisms, assessing the performance of porous BN compared to benchmarks in the field and outlining ways to improve the adsorption performance further.

## Introduction

Chemical separations account for more than half of the total energy consumed in industry owing to the many separation processes performed *via* distillation (Sholl and Lively, 2016). Adsorbent materials or membranes, which are able to separate molecules based on size exclusion principles and/or chemistries, offer a possibly less energy intensive and hence more sustainable route to separations (Sholl and Lively, 2016). Realizing the potential of adsorption and membrane separation requires the production of porous materials with tailorable properties and porosities. Today, research in this area primarily focuses on materials such as metal-organic frameworks (James, 2003), zeolites (Misaelides, 2011), carbon-based materials (Dias et al., 2007), covalent organic frameworks (Côté et al., 2005), and polymer of intrinsic microporosity (McKeown and Budd, 2006). A recent addition to this list is porous boron nitride (BN). Owing to its high thermal stability, “rich” chemistry, and high surface area [up to ~2,000 m^2^ g^−1^ reported so far (Li et al., 2013b; Marchesini et al., 2017b)], porous BN has been tested for a range of different applications such as catalysis (Venegas et al., 2017), CO_2_ capture, oil spill clean-up (Lei et al., 2013), hydrogen storage (Portehault et al., 2010; Lei et al., 2014; Weng et al., 2014), and water cleaning (Zhang et al., 2012; Li et al., 2013a,b; Lei et al., 2015). Porous BN is somewhat analogous to activated carbon in terms of its turbostratic to amorphous structure with 6-member rings of alternating B and N atoms. Yet, its chemistry is obviously different from that of carbonaceous materials as the material is made of polar B-N bonds. BN materials are typically hydrophobic. Such a feature can be particularly advantageous for the adsorption of organics, either in the vapor phase or as part of a water/organic mixture in the liquid phase. In fact, a number of studies report the use of porous BN for the removal of organic molecules (e.g., dyes, pharmaceutical molecules, oils) from water Zhang et al., 2012; Li et al., 2013a,b; Lei et al., 2015. The sorption mechanisms are typically associated to: (i) π-π stacking interactions between the BN sheets and aromatic dye molecules (Zhang et al., 2012; Liu et al., 2014, 2015); (ii) electrostatic dipole-dipole interactions between the polar B-N bond and the polar dyes (Liu et al., 2014; Lin et al., 2016); (iii) physisorption in the micropores of BN (Liu et al., 2014; Lin et al., 2016), and (iv) hydrophobic interactions (Yu et al., 2018). The adsorption capacity can be highly dependent upon the pH of the solution in the case of dye sorption, due to electrostatic interactions with the polar dyes, which are weakened by competitive adsorption of protons or hydroxides (Singla et al., 2015; Li et al., 2016). For a similar reason, OH-functionalised BN adsorbs more cationic dyes (e.g., methylene blue) than its pristine counterpart (Li et al., 2015). Porous BN materials exhibit competitive sorption capacities of selected organics compared to other common adsorbents such as activated carbons (Yu et al., 2018). Interestingly, many studies on the removal of organics from water using porous BN, report multiple adsorption and desorption cycles with minimal (5–15%) decrease in performance (Li et al., 2013b, 2015; Lei et al., 2015; Zhao et al., 2015; Liu et al., 2016). This behavior is surprising given the demonstrated instability of some porous BN structures in the presence of water, which causes a decrease in porosity (Shankar et al., 2018). This raises a question around the effect of BN porosity in these applications. Overall, the studies above serve as proof-of-concept for the use of porous BN as adsorbent for a given organic. Today though, the materials parameters—i.e., surface area, pore network, chemistry—that dictate adsorption of specific organics remain vastly unknown.

Herein, we synthesized BN samples exhibiting a range of pore structures and crystallinity as well as distinct chemistries (Marchesini et al., 2017a; Shankar et al., 2018) The pore structure of the materials was controlled *via* manipulation of the precursor type and ratio used to synthesized BN (see our previous study Marchesini et al., 2017a). We tuned the crystallinity and chemistry—particularly the amount of O groups—using the synthesis temperature (Shankar et al., 2018). We tested all the porous BN samples for the adsorption of different organic molecules, either directly, through vapor sorption analyses or as part of a water/organic mixture in the liquid phase. The organics selected for this study cover different classes of chemicals in terms of polarity and molecular size: alkanes (linear and branched), cycloalkanes, aromatics alcohols, and dyes. We characterized the samples before and after adsorption and linked their materials features to the adsorption behaviors.

### Materials Synthesis

We synthesized porous BN materials following a procedure previously developed by our group Marchesini et al., 2017b. Boron- and nitrogen- containing precursors were mixed and ground in different molar ratios. The precursors were placed in an alumina boat crucible and heated up to the 1050 or 1500°C for 3.5 h (10°C/min ramp rate) under nitrogen gas flow (50 cc/min during analysis, 3 h at 250 cc/min to purge). The furnace was cooled while flowing nitrogen until ambient temperature. We refer to porous BN samples synthesized at 1050°C as “low temperature BN” samples, and to porous BN samples synthesized at 1500°C as “high temperature BN” samples. We used three different sample formulations as follows:

- BN-U5: molecular ratio of urea:boric acid = 5:1;
- BN-MU0.25:5: molecular ratio of melamine:urea:boric acid = 0.25:5:1;
- BN-MU1:5: molecular ratio of melamine:urea:boric acid = 1:5:1.

### Materials Characterization

Materials characterization techniques were used to: (i) verify that the synthesized samples cover a range of porosity, crystallinity and chemistry as envisioned initially and (ii) assess the impact of adsorption on the materials features and (iii) extract information of the adsorption mechanisms.

#### Structural Properties and Morphology

Nitrogen physisorption isotherms were measured using a porosity analyser (Micromeritics 3Flex) at −196 °C. Prior to nitrogen sorption analysis, the samples were degassed overnight at 120 °C at roughly 0.2 mbar pressure and degassed again *in-situ* on the porosity analyser for 4 h down to around 0.003 mbar at 120 °C. From the nitrogen isotherms, we derived the surface areas, total pore volumes, micro- and mesopore volumes of BN samples and pore size distributions (Rouquerol et al., 2013). The surface areas of the samples were calculated using the Brunauer-Emmett-Teller (BET) method (Brunauer et al., 1938). The total volume of pores was calculated from the volume of N_2_ adsorbed at P/P_0_ = 0.97. The micropore volume was determined using the Dubinin Radushkevich method (Chen and Yang, 1994). The pore size distributions were calculated using a DFT model for carbons with slit-shape pores using the Micromerics software.^1^ We assessed the crystallinity (or lack of) of our samples *via* powder X-ray diffraction (XRD) using an X-ray diffractometer (PANalytical X'Pert PRO) in reflection mode. The operating conditions included an anode voltage of 40 kV and an emission current of 40 mA using monochromatic Cu Kα radiation (λ = 1.54178 Å).

#### Chemical Properties

The porous BN samples were characterized by Fourier Transform Infrared (FT-IR) spectroscopy. The samples were first ground in an agate mortar and spectra were collected in the range of 600–4000 cm^−1^ using a Perkin-Elmer Spectrum 100 Spectrometer equipped with an attenuated total reflectance (ATR) accessory. X-ray Photoelectron Spectroscopy (XPS) was performed using a Thermo Scientific K-Alpha^+^ X-ray Photoelectron Spectrometer equipped with a MXR3 Al Kα monochromated X-ray source (hν = 1486.6 eV). X-ray gun power was set to 72 W (6 mA and 12 kV). All high resolution spectra (B 1*s*, N 1*s*, C 1*s*, and O 1*s*) were acquired using 20 eV pass energy and 0.1 eV step size. The samples were ground and mounted on the XPS sample holder using conductive carbon tape. Thermo Avantage software (ThermoFisher Scientific) was used to analyse the data. The XPS spectra were shifted to align the peak for adventitious carbon (C-C) at 285.0 eV.

### Materials Testing

#### Organic Vapor Sorption

We conducted these analyses to assess the “idealized” (i.e., single adsorbate, no competitive adsorption) adsorption of several molecules with different polarities and molecular sizes. We performed vapor sorption experiments on an IGA gravimetrical analyser (Hiden Isochema). The IGA analyser is equipped with a microbalance with ± 1 μg resolution. The samples (~30 mg) were degassed *in-situ* at 120 °C for 4 h using a furnace attachment, prior to analysis. An ultra-high vacuum of up to 10^−6^ mbar could be created inside the chamber. Solvents were purified by cycles of evacuation followed by vapor equilibrium, repeated for at least 10 times. The solvents were vaporized and dosed up to 90 % of their saturation pressures at 25 °C: n-heptane (P_0_: 61.12 mbar), 2,3-dymethylpentane (P_0_: 91.82 mbar), toluene (P_0_: 37.93 mbar), methylcyclohexane (P_0_: 61.79 mbar), 1-butanol (P_0_: 9.22 mbar), methanol (P_0_: 169.24 mbar). The temperature was controlled using an external water bath equipped with a thermocouple which maintained a temperature stability of ± 0.3 °C. Equilibrium for each pressure point was predicted by the IGA software after a minimum wait of 20 min, by checking for weight changes using the least squares regression to extrapolate the asymptote (tolerance of 99 %). Pressure increase/decrease was performed with a rate of ~0.25–1 of the step pressure change. All solvents were of analytical grade. We performed *in situ*-degas in between each experiment at 250 °C to regenerate porous BN. Solvent selectivity was calculated as the ratio of quantity adsorbed of two pure solvents measured under the same conditions, assuming equal partial pressures.

#### Water Vapor Sorption

Prior to testing the removal of organics from water, we assessed water sorption to evaluate the affinity of the samples with water and quantify their water adsorption capacity. Porous BN samples were degassed at 120 °C at roughly 0.2 mbar pressure and again *in-situ* on the porosity analyser (Micromeritics 3Flex) for 4 h down to around 0.003 mbar. We collected water vapor isotherms at 30 °C for porous BN samples at up to 90% relative humidity in a gas/vapor volumetric analyser.

#### Organics Removal From Water

We used a dye [Rhodamine B, RhB (HPLC, Sigma Aldrich)] as a representative organic molecule owing to the ease of monitoring dye sorption using UV-vis spectroscopy. In a typical test, 250 mL of stock solution (of 40 mg/L in deionized water) were vigorously stirred and 100 mg of porous BN powder were added to the solution while stirring. At 0, 5, 10, 30, 60, 120, 240 min, about 2 mL of solution were collected with a syringe and filtered with a syringe filter (0.45 μm) to remove BN powder. We then analyzed these solutions using UV-vis spectrophotometry to determine the dye concentration. UV-vis spectra were collected between 400 and 650 nm (λ_maxRhodamine_ = 554 nm) at a scan speed of 480 nm/min using a Perkin Elmer Lambda 35 spectrophotometer with quartz cuvettes. Calibration was performed by preparing solutions with decreasing dye concentrations (see calibration curve in Supplementary Figure 1, R^2^ > 0.99). After 240 min, BN samples were filtered using a Buchner filtration system, dried in the oven at 120 °C and then heated at 600 °C (10 °C/min) for 3 h in a tubular furnace under air gas flow (50 mL/min) before being reused for a second adsorption cycle. We selected this regeneration temperature based on the decomposition temperature of the dye, as determined by TGA analysis (Netzsch TG209 F1 Libra, 10 °C/min ramp rate, under air gas flow at 0.1 L/min; Supplementary Figure 2A).

## Results and Discussion

**Figure 1:**
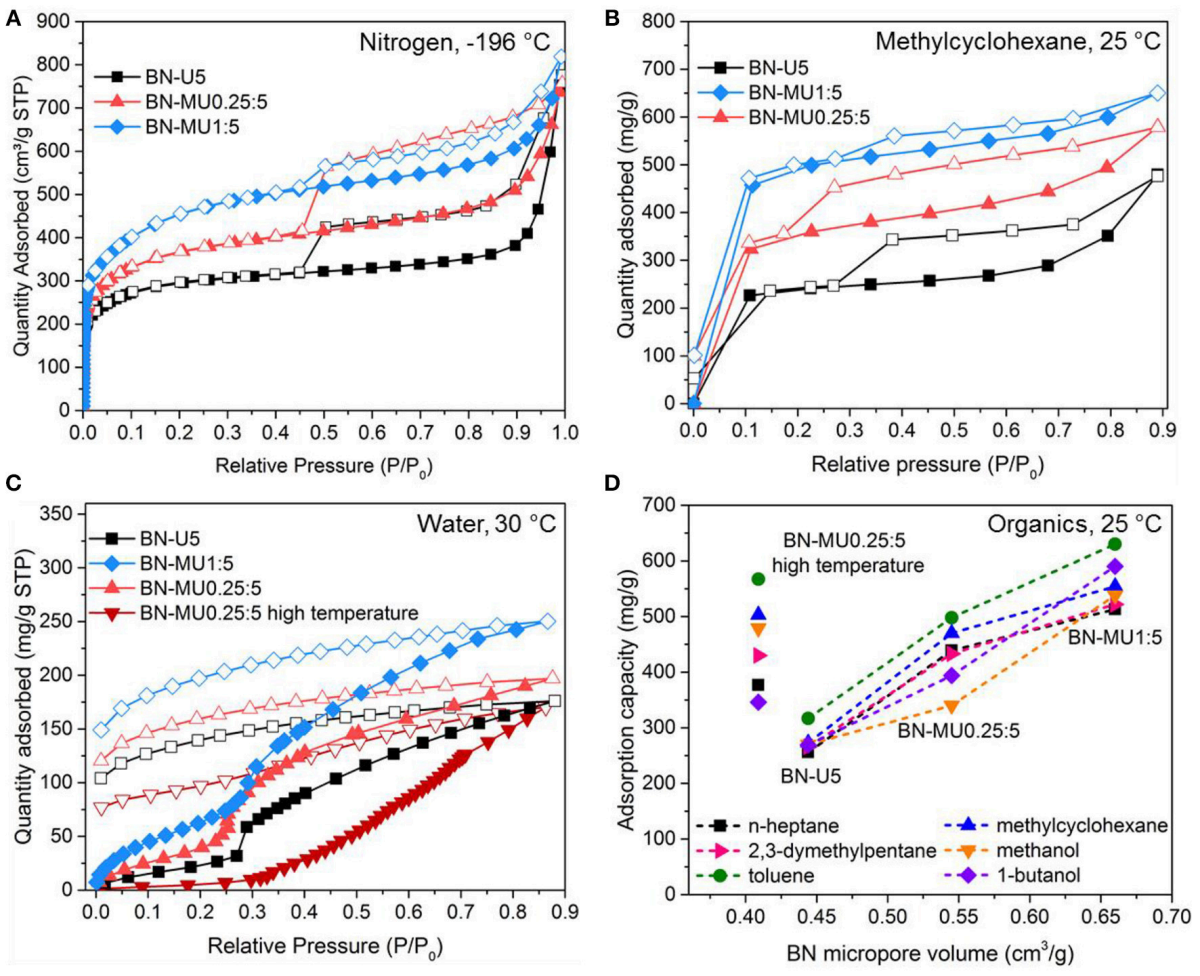
Organics and water sorption behaviors of porous BN samples of varying pore structures: **(A)** N_2_ sorption isotherms at −196 °C for three low temperature porous BN samples, **(B)** methylcyclohexane vapor sorption isotherms for the same three low temperature porous BN samples at 25 °C, **(C)** water vapor sorption isotherms for low temperature porous BN and one high temperature porous BN sample at 30 °C, **(D)** organic vapor sorption capacity at 0.6 P/P_0_ vs. micropore volume of porous BN for three low temperature samples and a high temperature BN. Open symbols in the figures represent desorption.

First, we confirmed the “profiles” of the materials synthesized using a number of characterization techniques to assess the range of porosity, crystallinity, and functionalization. As expected, the porous BN materials were all micro/mesoporous but displayed different pore volumes and BET surface areas (Table 1; Figure 1A). The samples covered a range going from 734 to 1650 m^2^g^−1^ in terms of surface area and 31 to 62% in terms of microporosity volume compared to total porosity. For all samples, the surface area and porosity decreased upon thermal treatment, as expected (Shankar et al., 2018). All samples contained oxygen atoms as part of their structure, as determined from XPS analyses (Table 1) and high temperature porous BN samples generally exhibited a lower oxygen content compared to low temperature porous BN samples. All porous BN samples displayed very similar XRD patterns with the same peak position for the (002) peak between the different samples, indicating similar d (002)-spacing (~0.35 nm) (Supplementary Figures 3A,B). The absence of other reflections other than the (002) and (100) and the large width and low intensity of the peaks confirmed that all samples were turbostratic. Despite a similar lack of crystallinity in the bulk phase between the low and high temperature BN samples, our prior work identified localized regions of increased crystallinity and thereby hydrophobicity in the high temperature BN sample (Shankar et al., 2018). We assessed the effect of these crystalline and hydrophobic regions on the adsorption behavior below.

**Table 1:** Overview of the porosity and chemistry of the porous BN samples: textural parameters derived from nitrogen sorption isotherms at −196 °C and relative atomic percentages derived from XPS analysis.

**Sample**	**S_**BET**_** **(m^**2**^/g)**	**V_**tot**_** **(cm^**3**^/g)**	**V_**micro**_** **(cm^**3**^/g)**	**V_**meso**_** **(cm^**3**^/g)**	**V_**micro**_/V_**tot**_** **(%)**	**O 1*s*** **(at %)**	**N 1*s*** **(at %)**	**B 1*s*** **(at %)**
**LOW TEMPERATURE BN**								
BN-U5	1,092	0.927	0.444	0.483	48	7	41	52
BN-MU0.25:5	1,339	1.026	0.545	0.481	53	8	40	52
BN-MU1:5	1,650	1.120	0.660	0.460	41	9	39	52
**HIGH TEMPERATURE BN**								
BN-U5	734	0.999	0.305	0.694	31	6	42	52
BN-MU0.25:5	940	1.095	0.409	0.686	62	6	42	52
BN-MU1:5	980	1.005	0.460	0.545	54	4	43	53

We then tested the low temperature porous BN materials (different pore structures and similar chemistries) for adsorption of C-7 hydrocarbons exhibiting different sizes/shapes (e.g., linear n-heptane or branched 2,3-dimethylpentane) and/or chemistries (e.g., aromatic toluene or non-aromatic methylcyclohexane) and two different alcohols (methanol and 1-butanol). Through this study, we intended to identify links between the materials physical properties, including porosity, and the adsorption performance. The results are presented in Figure 1B for methylcyclohexane and in Supplementary Figure 4 for all other chemicals. The three samples exhibited different sorption isotherms. Given that their chemical composition was similar, the differences are attributed to differences in physisorption dictated by the pore structure of the materials. In fact, the organic vapor sorption isotherms followed the same patterns as the nitrogen sorption at −196 °C (compare Figures 1A,B). The total vapor sorption capacity increased with increasing surface areas and most of the vapors were adsorbed at low relative pressures, in the microporous regions. The presence of mesopores caused capillary condensation (e.g., see BN-MU0.25:5) as indicated by the presence of hystereses in the desorption curves. Most of the vapors desorbed (Figure 1B) as the pressure was lowered, confirming that little/no chemisorption occurred.

We also evaluated water sorption for different low temperature porous BN samples at 30 °C (Figure 1C). Water uptake increased with the surface areas. Importantly, the water did not completely desorb upon reducing the pressure as observed from the “open” hystereses. As highlighted in a previous study (Shankar et al., 2018), this behavior originates from the hydrolysis of the BN materials through the following reaction: 2*BN*+3*H*_2_*O*→*B*_2_*O*_3_+2*NH*_3_. The hydrolysis of porous BN takes place due to the presence of defects in the structure (Motojima et al., 1982; Alkoy et al., 1997; Streletskii et al., 2009; Cao et al., 2012; Shankar et al., 2018). Upon thermal treatment, as mentioned above, we form localized regions of increased crystallinity and hydrophobicity. Hence, the high temperature porous BN showed a reduced water uptake capacity, especially at low relative pressures (Figure 1C). At higher pressures, the remaining defects sites react with water leading to decomposition of the material (Shankar et al., 2018).

From the organic vapor sorption isotherms, we calculated the sorption capacity at 0.6 P/P_0_ (Figure 1D). At this relative pressure, organic vapor uptake had ceased and BN materials were saturated. Sorption capacities of low temperature porous BN samples increased with increasing micropore volumes for all organics considered (Figure 1D). We found a linear trend between BN micropore volume and sorption capacity for porous BN materials with similar chemistries. This aligns with the behavior observed in activated carbons (Lillo-Ródenas et al., 2005). All porous BN materials adsorbed more toluene than any of the other tested adsorbates. We hypothesized that this is due to the presence of π-π interactions between the *h*-BN rings in porous BN and the aromatic rings in toluene, which contribute to improved physisorption. To put the results in perspective, porous BN exhibited toluene and methanol sorption capacities of the same order of magnitude to activated carbons with similar surface areas (toluene: 700 vs. 950 mg g^−1^; methanol: 600 vs. 800 mg g^−1^) (Xu et al., 2015). While the materials absorbed large amounts of organics, they were not selective (Supplementary Figure 5; linear n-heptane vs. branched 2,3-dimethylpentane; aromatic toluene vs. non-aromatic methylcyclohexane). This is related to the multimodal pore size distribution of the material—i.e., no narrow pore size distribution—that prevent size exclusion (Supplementary Figure 6). Similarly, we tested a high temperature porous BN sample for organic vapor sorption to evaluate the effect of the localized hydrophobic regions of the material (Figure 1D). Despite its lower porosity compared to the low temperature BN samples, the high temperature BN sample exhibited increased uptake of all organics. Further support for this observation is provided below when investigating dye sorption.

**Figure 2:**
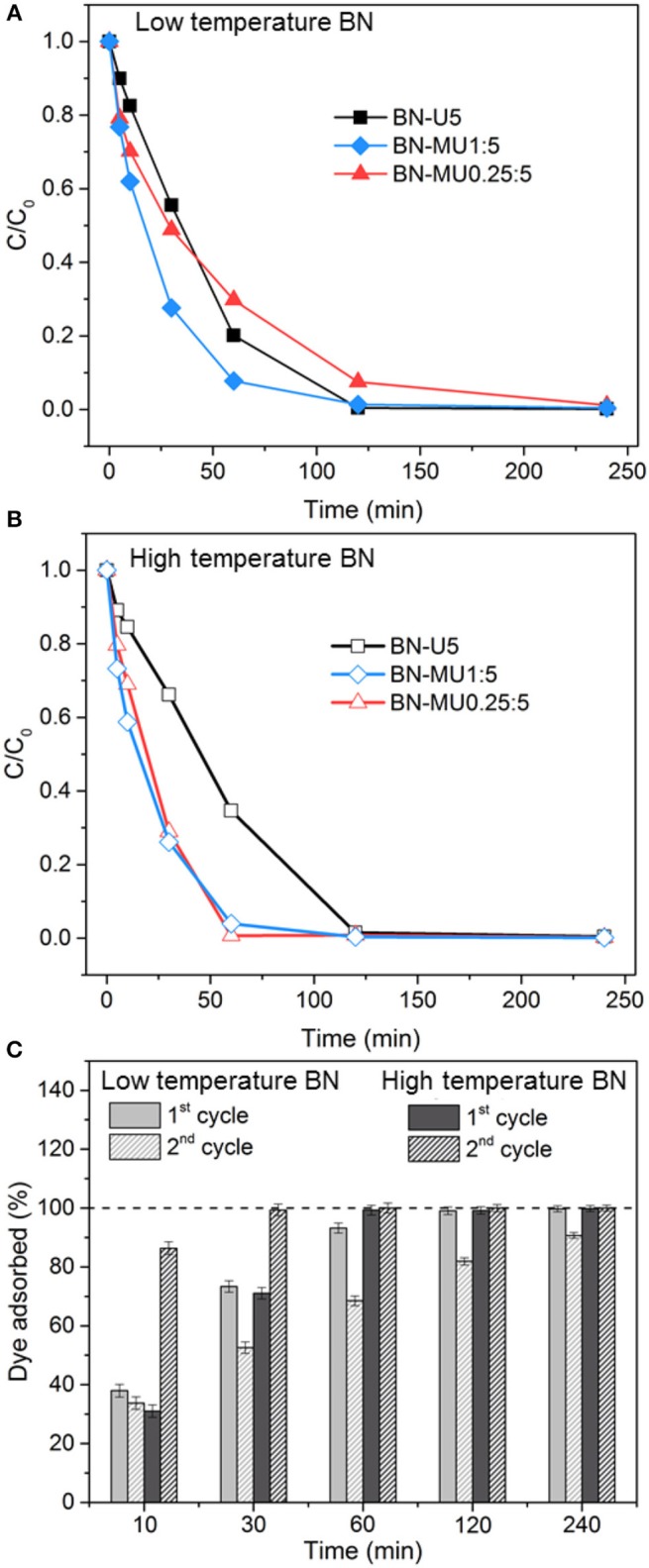
Removal of an organic (Rhodamine B) from water using porous BN samples: **(A)** RhB sorption over time for the low temperature porous BN samples, **(B)** RhB sorption over time for the high temperature porous BN samples, **(C)** evaluation of the recyclability of the best performing porous BN samples at low (BN-MU1:5) and high (BN-MU0.25:5) temperature after regeneration in air at 600 °C.

The adsorption potential of all porous BN materials was finally evaluated for a practical application. We employed the porous BN samples (low and high temperatures) for the removal of organics (here Rhodamine B, RhB) from water. The results are presented in Figure 2. All samples achieved complete removal of the dye within 4 h or less (Figures 2A,B). The time needed to reach complete removal reduced or remained unchanged (for BN-U5) when using the high temperature samples despite their initially lower surface area. Again, high temperature porous BN materials displayed a higher adsorption capacity for organics. We attribute this observation to the increased hydrophobicity and water stability of the high temperature BN samples, as well as to the presence of a larger quantity of π electrons, which allowed greater affinity for the adsorbate while preventing *in-situ* collapse of the material.

We regenerated the better performing low and high temperature samples (BN-MU1:5 and BN-MU0.25:5, respectively) at 600 °C in air and tested them for a second adsorption cycle. The results are reported in Figure 2C. While the low temperature BN sample exhibited a decreased capacity after regeneration (10 % decrease at 4 h), the high temperature BN sample maintained its original capacity. This is related to the greater stability of the high temperature sample in the aqueous environment compared to the low temperature sample. Analyses are presented below to confirm this aspect. Interestingly, the high temperature porous BN sample adsorbed more dye in the 2nd cycle than in the 1st cycle for the same exposure time. We explain this by the greater dispersibility of regenerated sample. Indeed, after regeneration and prior to the second sorption run, the sample exhibited finer particles.

We characterized the BN samples after the 1st cycle of dye removal from water and subsequent regeneration in air at 600 °C. This was done to confirm whether a collapse (partial or complete) of the porosity occurred and influenced the adsorption in the 2nd cycle as observed above. To quantify the impact of this potential collapse on the adsorption behavior, both the low and high temperature BN samples were analyzed—the high temperature BN samples being *a priori* less prone to collapse. We note that TGA and XPS analyses confirmed that the dye was completely removed upon regeneration in air at 600 °C (Supplementary Figure 2): no carbon residues were observed after regeneration.

**Figure 3:**
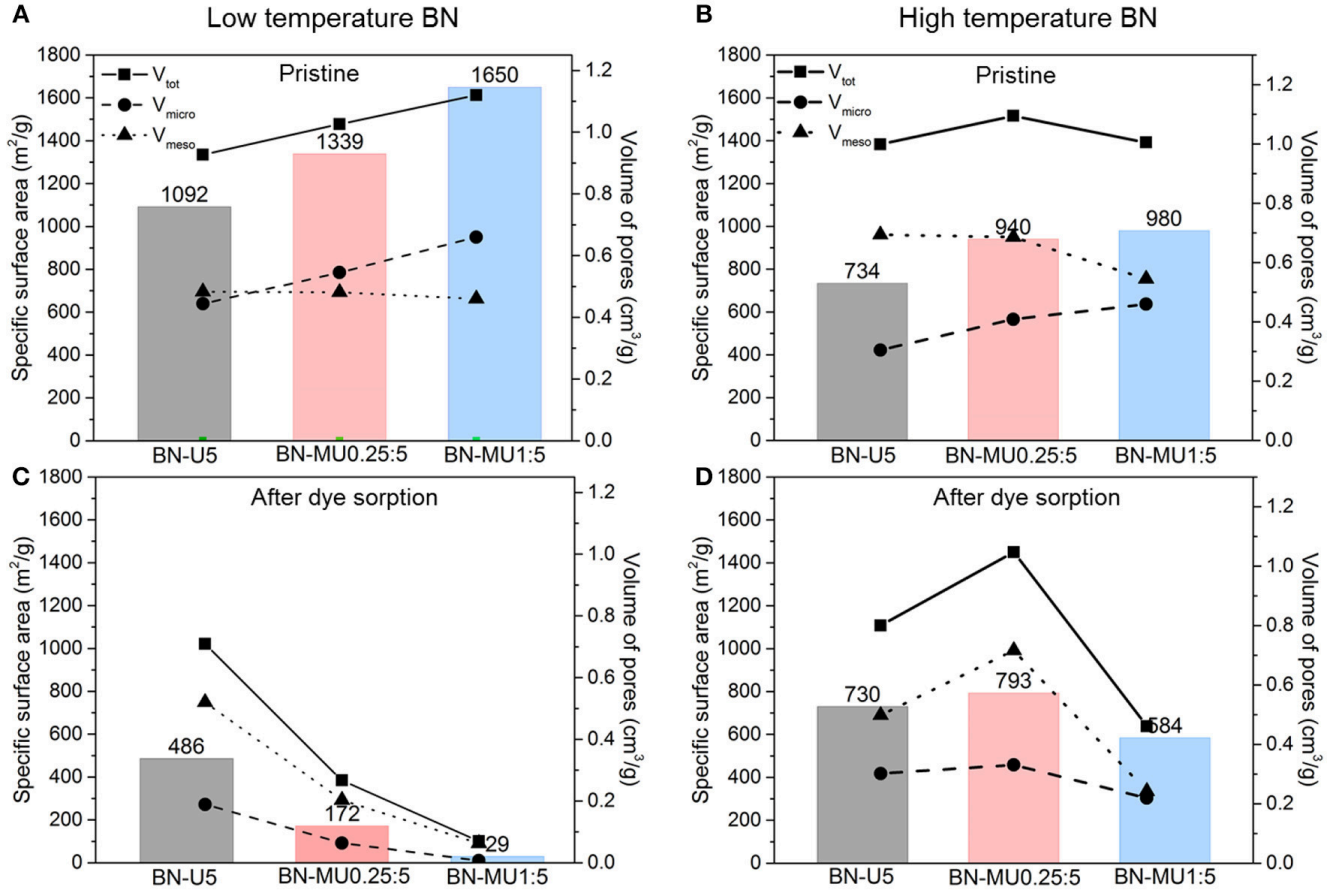
Changes in the porosity of the BN samples upon adsorption and regeneration at 600 °C in air. Textural parameters derived from N_2_ sorption isotherms at −196 °C for porous BN samples: **(A)** synthesized at low temperature, **(B)** synthesized at high temperature, **(C)** synthesized at low temperature after dye sorption and regeneration, **(D)** synthesized at high temperature after dye sorption and regeneration.

First, we analyzed the changes in porosities of the BN samples before testing and after regeneration (Figure 3). All low temperature porous BN samples showed a significant decrease in surface area and micropore volume after regeneration pointing to a collapse of the structure *via* hydrolysis (Shankar et al., 2018). Interestingly, the percentage of micropores decreased significantly in all porous BN samples, indicating that the micropores were initially most susceptible to hydrolysis. This hypothesis is supported by the fact that porous BN samples with higher micropore volumes (BN-MU1:5 > BN-MU0.25:5 > BN-U5), were more unstable, as indicated by a larger decrease in surface area and pore volume. For instance, highly microporous BN-MU1:5 exhibited a significant decrease in surface area from 1650 to 29 m^2^g^−1^, while the surface area of less microporous BN-U5 decreased from 1092 to 486 m^2^g^−1^. This is not surprising as the more porous samples are probably more defective and therefore more prone to hydrolysis. Despite the large decrease in surface area after regeneration, the RhB adsorption capacity only decreased by about 10 %. We hypothesized that the RhB concentration used was not high enough to saturate the adsorbent. Therefore, despite a reduced surface area after regeneration, the adsorption capacity was not significantly affected. The high temperature BN samples exhibited higher stability than the low temperature BN samples after testing and regeneration. Indeed, the samples maintained a high porosity after testing and regeneration. This aligns with the better performance of the high temperature BN samples observed before (Figure 2).

Continuing on the characterization of the materials post testing and regeneration, all samples showed a higher relative intensity of the (002)/(100) peaks, possibly indicating a preferred orientation of the crystals along the (002) plane (Supplementary Figure 3). In addition, the peak position for the (002) peak (~25°) shifted to higher angles, indicative of a decrease of the d(002)-spacing and the full width at half maximum (FWHM) decreased, demonstrating an increase in crystallite size (larger number of 2D layers). These three factors together showed that the samples exhibited higher crystallinity after exposure to water, probably as amorphous BN was more readily decomposed in water, resulting in an apparent increase in crystallinity.

**Figure 4:**
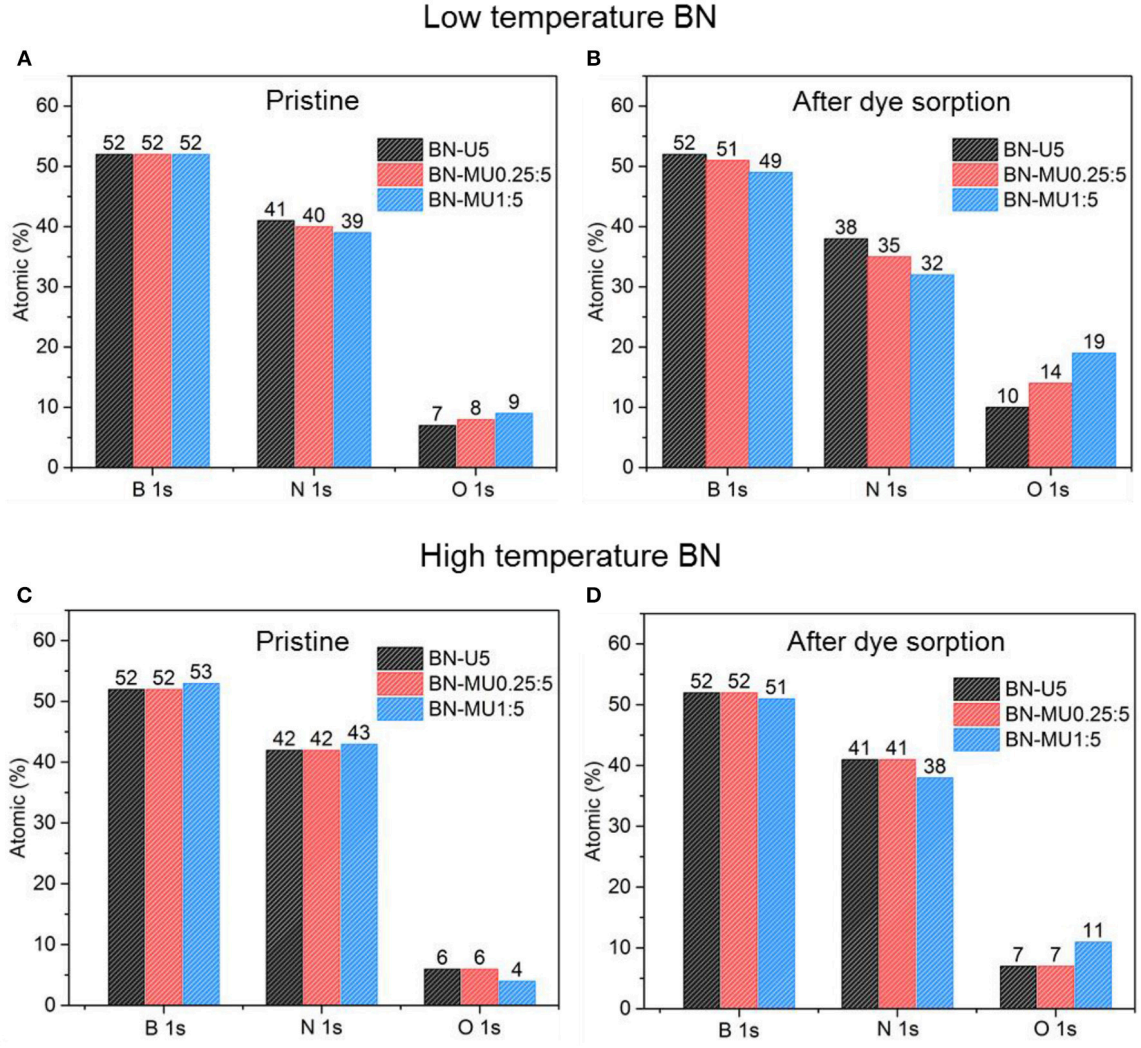
Changes in the chemistry of the BN samples upon adsorption and regeneration at 600 °C in air: **(A,B)** low temperature porous BN samples and **(C,D)** high temperature porous BN samples.

After demonstrating that the porosity of the high temperature BN samples was maintained upon adsorption testing and regeneration, we investigated any potential changes in the chemistry of the materials using XPS analyses. The relative atomic percentages derived from XPS confirmed that the oxygen content increased in all low temperature porous BN samples after adsorption testing and regeneration (Figures 4A,B). This further supports the hydrolysis of the low temperature BN samples, causing the oxidation of BN and formation of a larger quantity of boronoxynitride and B-OH groups (Lin et al., 2011). Samples with higher micropore volumes and surface areas (BN-MU1:5 > BN-MU0.25:5 > BN-U5) showed higher increases in oxygen content, indicative of more extensive hydrolysis, in line with the previously described structural changes. The relative nitrogen content decreased in all samples after exposure to water, confirming that a decomposition reaction is occurring, in which water reacts with BN to release ammonia: 3*H*_2_*O* + 2*BN* → *B*_2_*O*_3_ + 2*NH*_3_ (Lin et al., 2011). On the other hand, the high temperature porous BN samples did not show any major variations in oxygen content from XPS analysis for two of the samples (Figures 4C,D) (BN-U5-1500C and BN-MU0.25:5-1500C).

## Conclusions

We presented here a study on the adsorption behavior of porous boron nitride. For this, we synthesized porous BN samples with varied porosity, crystallinity and chemistry in order to identify the effect of these materials features on the adsorption of different organics. The main conclusions of this work are summarized below:

- Porous BN materials exhibit high sorption capacities for organics, as shown *via* gravimetrical vapor sorption analysis. The organic sorption capacity for toluene on porous BN (~650 mg/g at 25 °C and 0.6 P/P_0_) is comparable to the capacity of activated carbons previously reported in the literature;
- Porous BN materials exhibit low selectivity between the different organics tested here, as highlighted from similar adsorption capacities of organic vapors. This is attributed to their multimodal pore size distribution and amorphous nature, which prevent size exclusion separation.
- Porous BN materials demonstrate higher adsorption capacity of aromatic toluene compared to non-aromatic counterpart, methylcyclohexane. The enhanced toluene adsorption capacity is hypothesized to be due to π-π stacking interactions between the aromatic solvent and the B-N hexagonal rings.
- The adsorption of organics increases with the porosity when the chemistries of the materials are similar. Yet, porosity is not the dominating adsorption parameter. Increasing the crystallinity and hydrophobicity of porous BN materials *via* a heat treatment further allows enhanced organic removal from water, water stability and recyclability (2 cycles tested). The enhanced removal capacity was demonstrated from higher and more efficient removal of Rhodamine B dye from water. High temperature BN was also able to retain better the porosity and chemistry after regeneration. This is attributed to the reduced amount of defect sites that causes hydrolysis of the material and impairs its adsorption performance.
- Porous BN material can be used for organic adsorption from water, such as aromatic dyes (Rhodamine B) and shows complete removal and potentially good recyclability.

## Data Availability

All datasets generated for this study are included in the manuscript and the Supplementary Files.

## Author Contributions

SM and CP designed the experiments. SM and XW carried out the experiments. SM, XW, and CP analyzed the data. SM and CP wrote the manuscript. All authors have read and agreed the submission of the manuscript.

### Conflict of Interest Statement

The authors declare that the research was conducted in the absence of any commercial or financial relationships that could be construed as a potential conflict of interest.

## Footnotes

^1^http://www.nldft.com
