# Supplementary material for: Porous Boron Nitride Materials: Influence of Structure, Chemistry and Stability on the Adsorption of Organics
Source: Front Chem. 2019 Mar 26;7:160. doi: 10.3389/fchem.2019.00160 (PMC6443638; doi:10.3389/fchem.2019.00160)
Supplement: Supplementary file 1 [file Data_Sheet_1.docx]

Supplementary Material

Porous Boron Nitride Materials: Influence of Structure, Chemistry and Stability on the Adsorption of Organics

Sofia Marchesini, Xiyu Wang and Camille Petit^*^

Barrer Centre, Department of Chemical Engineering, Imperial College London, South Kensington Campus, London SW7 2AZ, UK

*** Correspondence:**Corresponding Author
[camille.petit@imperial.ac.uk](mailto:camille.petit@imperial.ac.uk)

Keywords: boron nitride, vapour sorption, adsorption, separations, water stability.

# Supplementary Figures


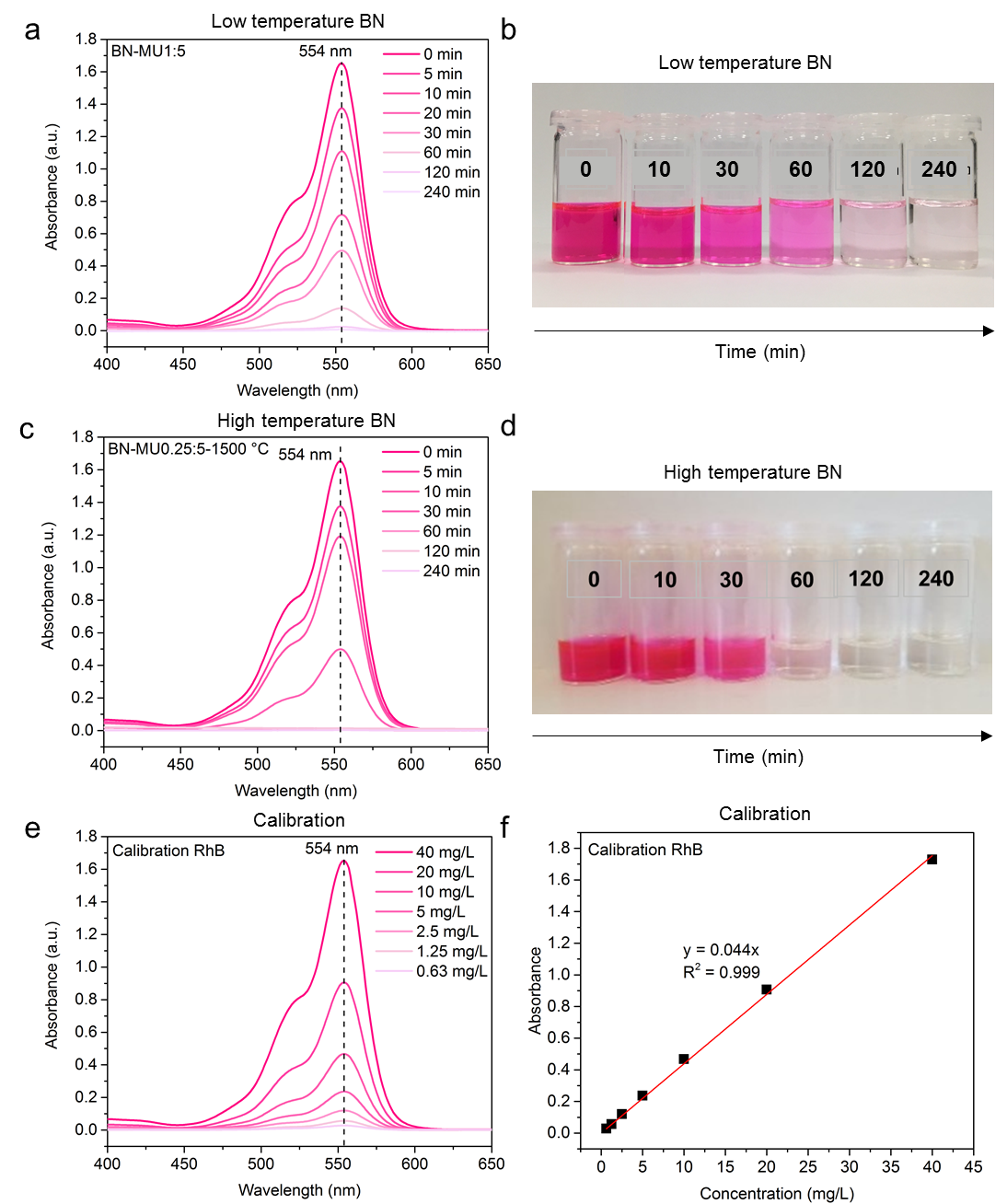


**Supplementary Figure 1.** Rhodamine B sorption capacity *vs* time showed in pictures and measured using UV-vis absorption spectroscope for: (a,b) low temperature porous BN and (c,d) high temperature porous BN. (e) UV-vis spectra for RhB solutions of known concentrations used for calibration, (f) calibration curve for RhB sorption data analysis.


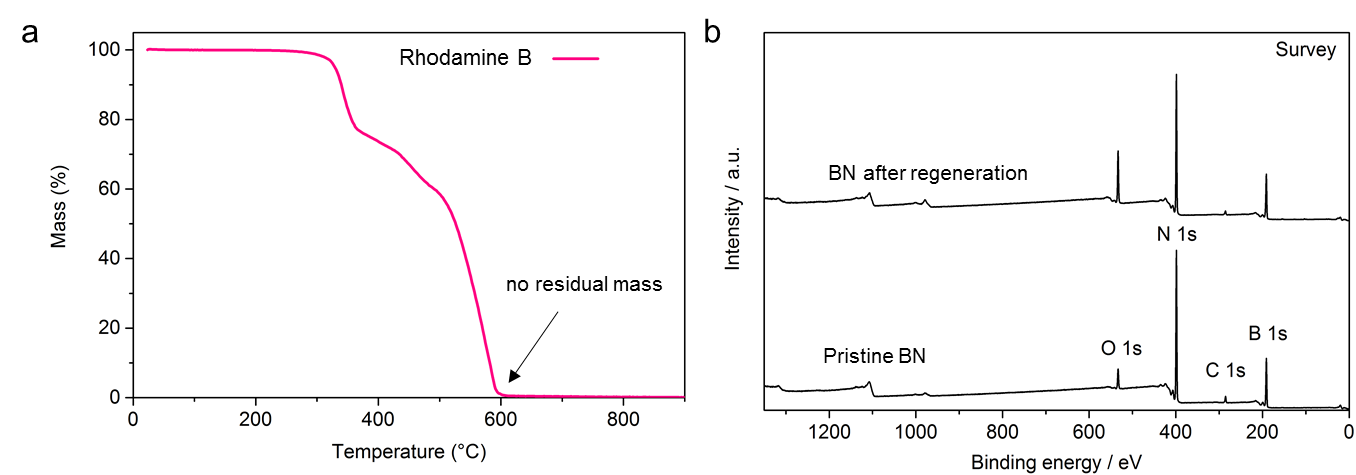
**Supplementary Figure 2.** (a) Thermogravimetric curve obtained for Rhodamine B in air showing full decomposition at 600 °C, (b) XPS Survey Spectra for porous BN before and after regeneration showing no increase in carbon content after regeneration.


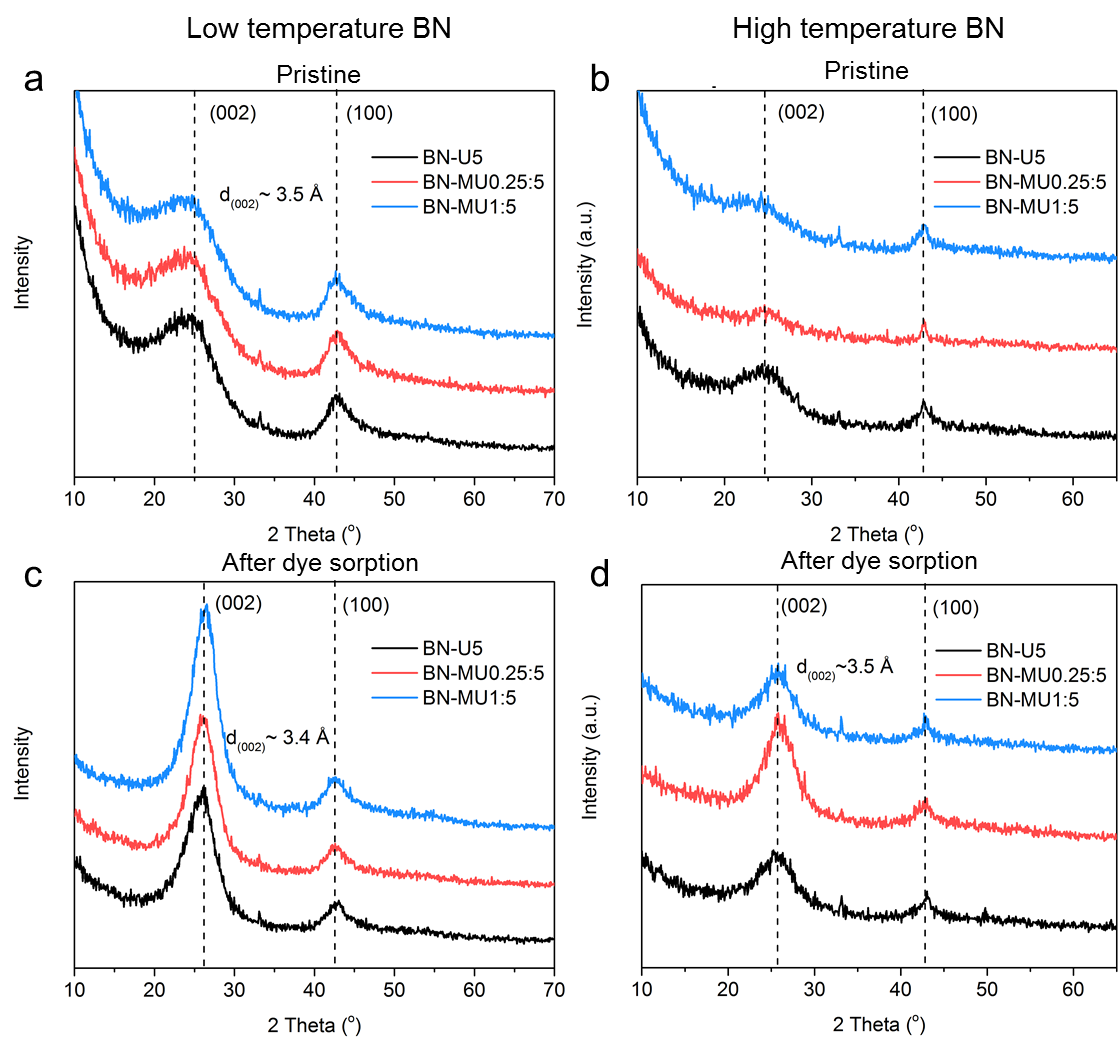


**Supplementary Figure 3.** XRD patterns for: (a,c) low temperature porous BN samples (a) pristine and (b) after dye sorption and regeneration at 600 °C in air; and (b,d) high temperature porous BN samples (a) pristine and (b) after dye sorption and regeneration at 600 °C in air.


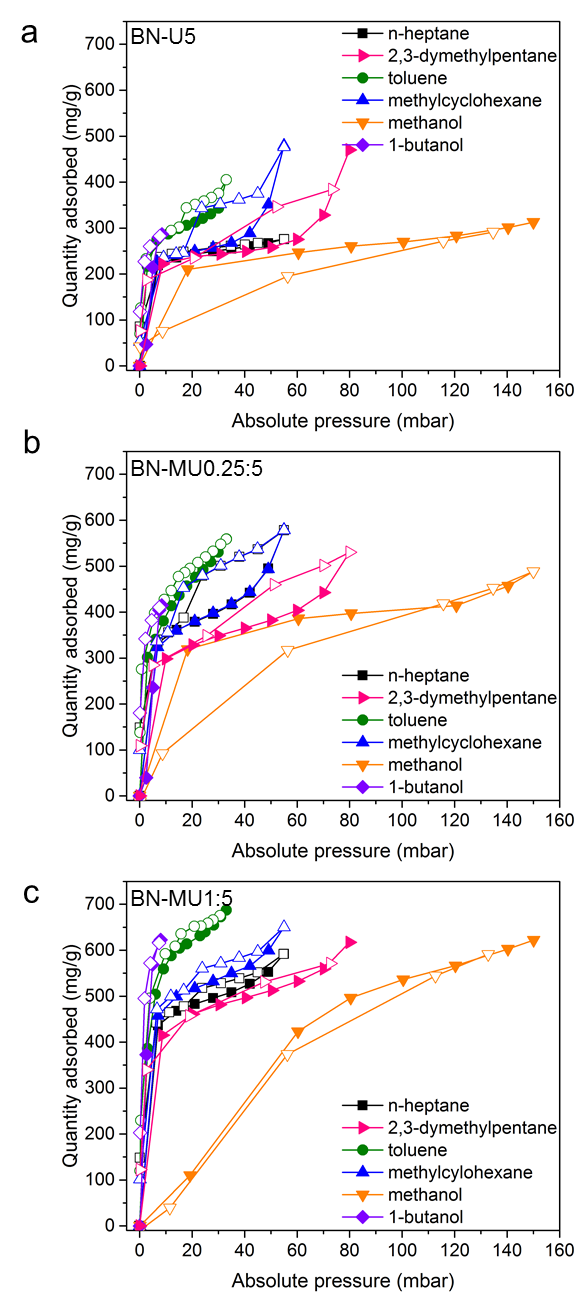


**Supplementary Figure 4.** Organic vapour sorption isotherms for different low temperature porous BN samples.

**
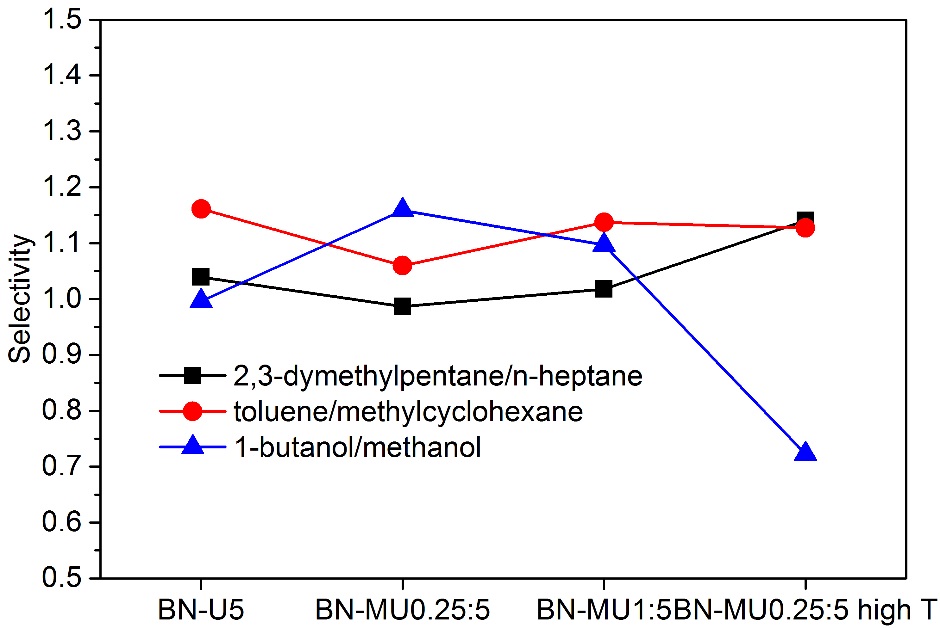
**

**Supplementary Figure 5.** Organic vapour sorption selectivities for different porous BN samples, calculated from the ratio of adsorption capacities at 0.6 P/P_0_.


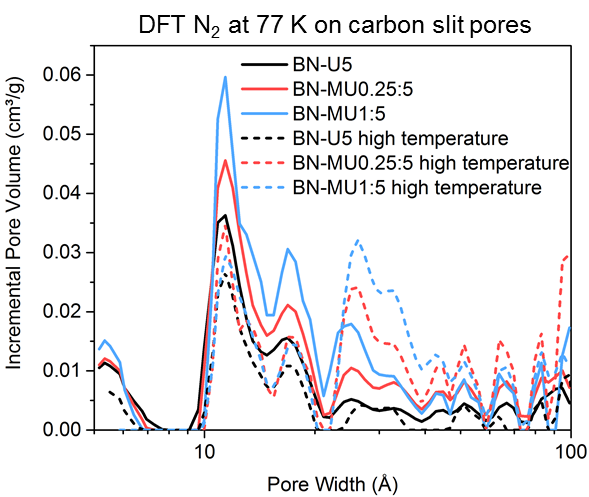


**Supplementary Figure 6.** Pore size distributions calculated from nitrogen sorption isotherms at 77 K using a DFT model for carbon with slit-shape pores on all porous BN materials.
